# Characterization of the complete mitochondrial genome of *Silurus soldatovi* in Korean river

**DOI:** 10.1080/23802359.2019.1687347

**Published:** 2019-11-08

**Authors:** Md. Jobaidul Alam, Mahmoud Aboalyamin Soliman Khalil, Kyung Su Kim, Sapto Andriyono, Chang Geun Choi, Hyun-Woo Kim

**Affiliations:** a Interdisciplinary Program of Biomedical, Mechanical and Electrical Engineering, Pukyong National University, Busan, Republic of Korea;; b KOICA-PKNU International Graduate Program of Fisheries Science, Graduate School of Global Fisheries, Pukyong National University, Busan, Republic of Korea;; c Gyeongsangnam-do Freshwater Fish Research Center, Miryang, Republic of Korea;; d Fisheries and Marine Faculty, Universitas Airlangga, Surabaya, Indonesia;; e Department of Ecological Engineering, Pukyong National University, Busan, Republic of Korea;; f Department of Marine Biology, Pukyong National University, Busan, Republic of Korea

**Keywords:** Next-generation sequencing, *Silurus soldatovi*, mitochondrial genome, alien species

## Abstract

The complete mitochondrial genome of *Silurus soldatovi* firstly collected from a native Korean river was determined by the bioinformatics assembly of the next-generation sequencing (NGS) reads. The circular mitogenome was 16,525 bp in length which harbored canonical 13 protein-coding genes, 22 tRNAs, and 2 rRNAs, which was identical to those of family Siluridae. Twenty-eight genes were located on H strand, whereas the remaining nine genes were on L strand. Except for COX1 gene (GTG), other 12 protein-coding genes were predicted typical start codons (ATG). Among the currently known mitogenome sequences, S*. soldatovi* showed highest identity (99.38%) to the Chinese haplotype of *S. soldatovi* (NC022723) followed by the Chinese haplotype of *Silurus asotus* (JX087351). Interestingly, intraspecies variations of *S. asotus* are higher than those of interspecies and further study should be made to elucidate the evolutional relationship between two *Silurus* species.

As the international trades of fish species increase, the risk of their harmful effects also grows when the non-native species are occasionally introduced to the native aquatic ecosystem. According to the Korean biodiversity database (https://species.nibr.go.kr/index.do), only two species in genus *Silurus* are currently reported; *Silurus asotus* and *Silurus microdosalis*. *Silurus soldatovi* is originally native in the Amur River basin in Russia and China (Wang et al. 2015). We here firstly report the complete mitochondrial genome of *S. soldatovi* collected from a tributary of Nakdong River, South Korea (E128°06′27.73″, N35°32′20.96″′) in 2018. The specimen and its DNA was stored at the Marine Biodiversity Institute of Korea (MABIK GR00002615). Its COI region showed the highest nucleotide sequence identity to *S. soldatovi* from China (AB860299; 99.61%) followed by *S. asotus* from China (JX087351, 97.03%). The complete mitochondrial genome of the specimen was determined by Illumina MiSeq system with the mitochondrial DNA isolated by a commercially available kit (Abcam, Cambridge, MA, USA). The TruSeq^®^ RNA library preparation kit V2 (Illumina, San Diego, CA, USA) was used with the fragmented mitochondrial DNA by Covaris M220 Focused-Ultrasonicator (Covaris Inc., Woburn, MA, USA). The complete circular DNA was constructed by the bioinformatic assembly of the raw reads using Geneious software version 11.0.2 (Biomatters Ltd., Auckland, New Zealand) (Kearse et al. 2012). The secondary structures of 22 tRNAs were predicted by ARWEN program (Laslett and Canbäck 2008).

The circular complete mitogenome of *S. soldatovi* (MN171302) was 16,525 bp in length, which consisted of 13 protein-coding genes, 22 tRNAs, and 2 ribosomal RNAs (12S and 16S). Twenty-one tRNAs were predicted to be folded into the typical clover-leaf structures, except for the tRNA-*Ser*. An unusual start codon was identified only in the COX1 gene (*GTG*), which is also shown in the other Silurid catfish (Alam et al. 2019). The incomplete stop codons (TA–/T–) were predicted in six genes including *ND2*, *COX2*, *COX3*, *ND3*, *ND4*, and *CYT b* genes.

A phylogenetic tree of the Silurid mitogenomes was constructed by the MEGAversion 7 program with minimum evolution algorithm (Kumar et al. 2016). Among the currently known mitogenomes in the GenBank database, *S. soldatovi* collected from Nakdong River showed the highest nucleotide sequence identity (99.38%) to the Chinese haplotypes of *S. soldatovi* (NC022723) followed by the Chinese haplotype of *S. asotus* (JX087351) with 97.17% identity (Figure 1). Interestingly, two Chinese haplotypes of *S. asotus* (JX087351 and JN116720) were more closely related to two *S. soldatovi* (MN171302 and NC022723) than their Japanese and the other Chinese haplotypes (JX256247 and NC015806). The genetic distance between Korean haplotype of *S. soldatovi* (MN171302) and Chinese haplotype of *S. asotus* (JX087351) was 0.032, whereas distance between two *S. asotus* from China (JX087351 and JX256247) was 0.054 exhibiting higher intraspecies variations. Further re-examination for those two species should be made to have a better understanding their evolutional relationship.

**Figure 1.**
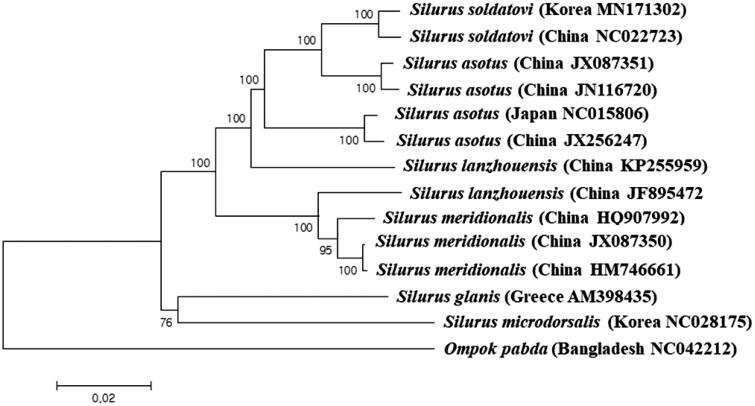
Phylogenetic relationship of *Silurus soldatovi* in the family Siluridae.

Phylogenetic tree was constructed with the currently reported complete mitogenomes in the family Siluridae by using the MEGA version 7.0 software by Minimum Evolution (ME) algorithm with 1000 bootstrap replications. GenBank accession numbers were shown followed by each species scientific name.
